# Comprehensive Numerical Analysis of Temperature Sensitivity of Spherical Microresonators Based on Silica and Soft Glasses

**DOI:** 10.3390/s23020717

**Published:** 2023-01-08

**Authors:** Maria P. Marisova, Alexey V. Andrianov, Elena A. Anashkina

**Affiliations:** Institute of Applied Physics of the Russian Academy of Sciences, 46 Ulyanov Street, 603950 Nizhny Novgorod, Russia

**Keywords:** microresonator with whispering gallery modes (WGMs), silica glass microsphere, tellurite glass microsphere, germanate glass microsphere, chalcogenide glass microsphere, thermo-optical effect, thermo-optical sensitivity

## Abstract

In recent years, the use of optical methods for temperature measurements has been attracting increased attention. High-performance miniature sensors can be based on glass microspheres with whispering gallery modes (WGMs), as their resonant frequencies shift in response to the ambient parameter variations. In this work, we present a systematic comprehensive numerical analysis of temperature microsensors with a realistic design based on standard silica fibers, as well as commercially available special soft glass fibers (GeO_2_, tellurite, As_2_S_3_, and As_2_Se_3_). Possible experimental implementation and some practical recommendations are discussed in detail. We developed a realistic numerical model that takes into account the spectral and temperature dependence of basic glass characteristics in a wide parameter range. To the best of our knowledge, spherical temperature microsensors based on the majority of the considered glass fibers have been investigated for the first time. The highest sensitivity *dλ*/*dT* was obtained for the chalcogenide As_2_Se_3_ and As_2_S_3_ microspheres: for measurements at room temperature conditions at a wavelength of *λ* = 1.55 μm, it was as high as 57 pm/K and 36 pm/K, correspondingly, which is several times larger than for common silica glass (9.4 pm/K). Importantly, *dλ*/*dT* was almost independent of microresonator size, WGM polarization and structure; this is a practically crucial feature showing the robustness of the sensing devices of the proposed design.

## 1. Introduction

Accurate temperature measurements are vital for many technological processes, various practical applications and scientific research. In recent years, in addition to non-optical sensors (liquid thermometers, bimetal thermometers, Peltier devices, resistance temperature detectors, and so on), optical temperature measurement tools have been increasingly used [1,2,3]. These photonic devices exploit the fact that their parameters may vary with temperature, thus affecting the properties of propagating light, such as wavelength, phase, intensity, and polarization [2]. Examples are stand-off thermometers, including radiation thermometers and pyrometers, Raman and Rayleigh scattering-based thermometers, thermometers based on spectral changes, and others [1,2].

Measurements with glass-fiber-based temperature devices are fairly common [4,5,6]. Solid microspherical resonators with whispering gallery modes (WGMs), which can be routinely manufactured by melting the tip of an optical fiber [3], form an important subclass of such systems. In general, these microspheres have a wide range of applications and can be produced from various materials using different techniques [7,8,9,10]. WGM-based temperature measurement devices attract a lot of attention due to the unique advantages of this platform: high sensitivity, small size (ranging from a few tens to a few hundred micrometers), light weight, wide temperature operating range, and lack of electromagnetic interference [7]. Microsphere thermo-optic sensors rely on two principles of operation: (i) a WGM resonant frequency drift due to the temperature dependence of refractive index and microresonator size and (ii) a luminescence peak shift in gain microspheres caused by changes in temperature [3,8]. This work is dedicated to the numerical study of spherical, fiber-based temperature microsensors that employ the first principle.

Modern technologies allow fabricating high-quality optical glasses with different chemical compositions and properties. Nowadays, silica glasses are by far the most common, owing to the advancement in fiber-optic communication systems. On the other hand, special soft glasses (chalcogenide, tellurite, germanate, fluoride, etc.) have immense potential thanks to their outstanding optical and physicochemical characteristics; the interest in their use has been growing steadily over the past few years. Various photonic devices based on these glasses offer enhanced performance compared to their silica-based counterparts; they are being actively studied in the frame of fiber, waveguide, and microresonator optics. Soft glasses usually have a much lower phonon energy and a wider transparency range than fused silica, thus allowing lasing in spectral regions that are unavailable for regular silica-based systems [11,12,13,14,15,16]. Chalcogenide and tellurite glasses exhibit enormous Kerr and Raman nonlinearities [17,18]. This makes them an exceptional platform for nonlinear wave conversion applications in a wide parameter range, including supercontinuum generation [17,19,20], Raman soliton generation [21], continuous-wave Raman generation [22], generation of optical frequency combs [23,24], all-optical switching [25], ultrafast metrology [26], sensing and biosensing [27], etc. Although the nonlinear optical processes are not directly related to temperature sensing, thermo-optical effects are crucial for their practical realization and efficient control [23]. Precise knowledge of the temperature response of a microresonator may help to advance the studies of nonlinear and laser effects in such systems [8,23]. The aforementioned applications, in turn, stimulate further improvements in fiber glass quality and properties, the optimization of technological processes and, most importantly, the launch of commercial products. Quite a lot of scientific institutes and laboratories are involved in the synthesis of special glasses and optical fiber fabrication; however, far from all of the interested researchers have access to their products. Currently, tellurite and germanate glasses can be manufactured on request. Chalcogenide glasses based on arsenic sulfide (As_2_S_3_) and arsenic selenide (As_2_Se_3_) are produced by different commercial companies, which undoubtedly greatly expands their availability.

Although wide-range mid-IR transparency and large Kerr and Raman nonlinear coefficients are irrelevant for temperature microsensor design, some of the considered special soft glasses have significantly larger thermal expansion coefficients than fused silica (almost 50 times higher for amorphous As_2_S_3_ and As_2_Se_3_ [28,29,30,31]), thus being interesting candidates for the development of highly sensitive thermometric devices.

In this work, we present a systematic comprehensive numerical analysis of temperature microsensors with a realistic design based on standard silica glasses, as well as special soft glasses (GeO_2_, tellurite, As_2_S_3_, and As_2_Se_3_), extensively discuss a possible experimental implementation, and formulate certain practical recommendations. The primary focus is on commercially available components for the operation at the light wavelength near 1.55 μm, hence combining the remarkable properties of soft glasses, the existing technological basis for microsphere fabrication from glass fibers [7], and the advantages of the well-developed, robust, and cheap telecommunication component base. We utilize a realistic numerical model that accounts for temperature- and spectral-dependent glass characteristics. To the best of our knowledge, the study of solid microspherical temperature sensors made of passive As_2_Se_3_, GeO_2_ and tellurite soft glass fibers has been performed for the first time.

## 2. Materials and Methods

Glass-based microresonators are routinely manufactured by melting the tip of an optical fiber: under the action of surface tension forces a solid sphere is formed [7]. This can be done, for example, using a CO_2_ laser for any type of glass [7,23,32]. A regular telecommunication fiber splicer may also be used to make fused silica microspheres [33], and a microheater to make soft glass microspheres [22,34]. Earlier, we successfully employed the latter method to produce different-sized spherical microresonators from commercially available chalcogenide and customized tellurite fibers [35,36].

**Figure 1:**
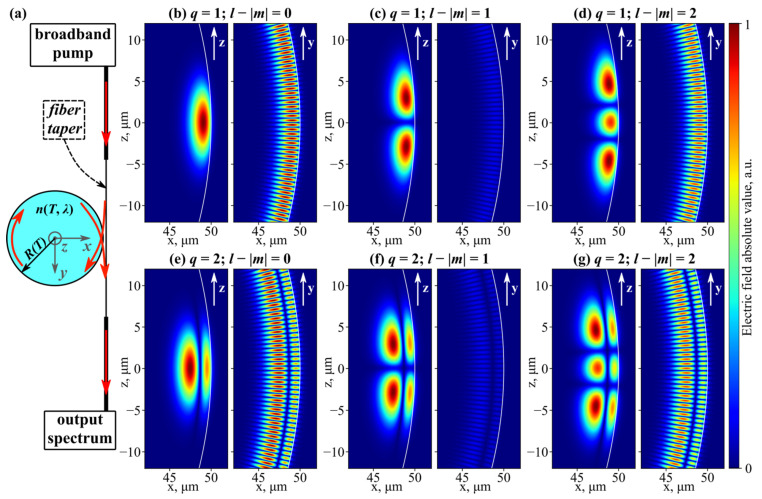
(**a**) Schematic of temperature microsensor; red arrows show light propagation direction. (**b**–**g**) Spatial distribution of electric field absolute value for WGMs with different indices (exact expressions are rather cumbersome and can be found in [38]). Eigenmodes shown in the same row have equal frequencies; color scale is identical for all figures. All calculations are for SiO_2_ microsphere with radius 50 μm.

The operation of temperature sensors based on microresonators with WGMs relies on the thermo-optical shift of resonant frequencies. A possible measurement scheme with such a microsensor is shown in Figure 1a. We assumed that the microsphere was pumped using a broadband source (for example, amplified spontaneous emission (ASE) from an Er-doped fiber); the input power was set to be low to avoid nonlinear Kerr effects. The light propagates along a thin fiber taper placed in the “equatorial” plane of the spherical microresonator (also denoted as *z* = 0). Regardless of the sphere material, a standard telecommunication silica fiber can be used to manufacture the microtaper [22,32,36,37], for example, employing a gas torch to soften and stretch the glass [33]. The suggested pumping setup facilitated the excitation of near-surface microsphere eigenmodes localized in the “equatorial” plane. The established coupling between the light inside the microtaper and the resonator optical modes was also used to extract and spectrally analyze the output radiation. In this configuration, the WGM frequencies appeared as spectral dips that were susceptible to variations in ambient parameters. The exact temperature change could be deduced from the corresponding eigenfrequency shift.

For a perfectly shaped solid dielectric microsphere, the WGM frequencies and electromagnetic fields can be found directly from the solution of Maxwell equations in a spherical coordinate system with definite boundary conditions [38]. Two eigenmode polarizations are possible (TE and TM); additionally, WGMs are enumerated with three integer indices: radial *q ≥* 1, polar *l ≥* 0, and azimuthal *–l ≤ m ≤ l* that define the spatial mode configuration. The number of electromagnetic field maxima can be expressed via *q*, *l*, and *m*: *q* in the radial direction, 2|*m*| in the “equatorial” direction (i.e., in the *z* = 0 plane), and *l* − |*m*| + 1 in the “meridional” direction, as illustrated in Figure 1b–g. Eigenmodes with *q* = 1, *l* = |*m*| are typically called fundamental WGMs.

The WGM eigenfrequencies were determined from the following characteristic equation [38]:

(1) $$\frac{{\left[{\left(nkR\right)}^{1/2}\;J_{\nu }\left(nkR\right)\right]}^{\prime }}{{\left(nkR\right)}^{1/2}\;J_{\nu }\left(nkR\right)}=n^{p}\frac{{\left[{\left(kR\right)}^{1/2}\;H_{\nu }^{\left(1\right)}\left(kR\right)\right]}^{\prime }}{{\left(kR\right)}^{1/2}\;H_{\nu }^{\left(1\right)}\left(kR\right)},$$

where *R* is the microsphere radius, *k =* 2*π/λ*, *f_l_^(0)^ = c/λ* is the eigenfrequency of a WGM with polar index *l*, *ν = l +* 1/2, *J_ν_* and *H_ν_*^(1)^ are Bessel and Hankel functions of the first kind of order *ν*, and *n*(*λ*) is the refractive index of the material, which for the considered glasses can be calculated from the Sellmeier equation (the used literature sources are provided in Table 1):

(2) $$n^{2}\left(\lambda \right)=A_{0}+\sum_{i=1}^{i=N}\frac{A_{i}\lambda^{2}}{\lambda^{2}-\lambda_{i}^{2}\;}$$

The radial mode index *q* corresponds to the sequence number of the root of the characteristic equation (1); whereas the azimuthal index *m* is not present in (1), as the WGM eigenfrequencies of an ideal microsphere are degenerate by *m*. Naturally, this degeneracy is lifted in a real experiment due to shape imperfections caused by the manufacture process. In the simplest case of a deformation into a spheroid with semiaxes *R_z_* and *R_x_* (maintaining axial symmetry along *z*) and the deformation parameter *η = (R_z_ − R_x_)/R*, the eigenfrequencies can be calculated as follows [39]:

(3) $$f_{l,m}=f_{l}^{\left(0\right)}\left(1-\frac{\eta }{3}+\frac{\eta m^{2}}{l\left(l+1\right)}\right)$$

**Table 1:** Glass parameters from the literature used in simulations. Numerical values are given at room temperature *T_0_* = 293 K and at light wavelength *λ* = 1.55 μm.

Glass	*n*(*λ*)	$\mathrm{Thermal}\;\mathrm{Expansion}\;\alpha =\frac{1}{L}\frac{dL}{dT}\;$		Thermo-Optic Effect *dn*/*dT*	
		*α*, 10^−6^ K^−1^	Available Data *α*(*T*)	$\frac{dn}{dT}$ 10^−6^ K^−1^	$\mathrm{Available}\;\mathrm{Data}\;\frac{dn}{dT}\left(T,\;\lambda \right)$
SiO_2_	[40]	0.48	*α*(*T*); 10 K ≤ T ≤ 300 K; [41]	8.2	$\frac{dn}{dT}\left(T,\;\lambda \right)$; 30 K ≤ T ≤ 300 K; [42]
GeO_2_	[40]	6.1	*α*(*T*); 95 K ≤ T ≤ 720 K; [43]	16	$\frac{dn}{dT}$ = *const*; [44]
TeO_2_−WO_3_− La_2_O_3_ (TWL)	[36]	14.2	*α*(*T*); T ≤ 600 K; [45]	−8	$\frac{dn}{dT}$ = *const*; [46]
As_2_S_3_	[47]	22.5	*α*(*T*); 2 K ≤ T ≤ 373 K; [28,29]	3.7	$\frac{dn}{dT}$(*λ*); [29]
As_2_Se_3_	[48]	21.4	*α* = *const*; 293 K ≤ T ≤ 423 K; [30,31]	48.6	$\frac{dn}{dT}$(*λ*); [31]

Thermally induced WGM frequency shifts mostly originate from two effects: thermal expansion of the resonator leading to changes in *R*, and refractive index dependence on temperature *n*(*T*), which is often referred to as the thermo-optic effect. For the examined glasses, these effects are usually considered to be linear at room temperature, and the corresponding coefficients are introduced: the thermal expansion coefficient *α* = *L*^−1^·*dL*/*dT* (*L* is the sample length) and the thermo-optic coefficient *dn*/*dT* (in absolute values). However, when exploring a broader parameter range, it is critical to account for the spectral and temperature dependency of *α*(*T*) and *dn*(*T*,*λ*)/*dT* as follows:

(4) $$R\left(T_{0}+\Delta T\right)=R_{0}(1+\int_{T=T_{0}}^{T=T_{0}+\Delta T}\alpha \left(T\right)dT)$$

(5) $$n\left(T_{0}+\Delta T,\;\lambda \right)=n\left(T_{0},\;\lambda \right)+\int_{T=T_{0}}^{T=T_{0}+\Delta T}\frac{dn}{dT}\left(T,\;\lambda \right)dT$$

where *T_0_* is the initial microsensor temperature (*T_0_* = 293 K), *R_0_* = *R*(*T* = *T_0_*) is the microsphere radius at room temperature, and Δ*T* is the temperature change. Unfortunately, the data required for the considered glasses are available in the literature only in a limited parameter range, which, however, contains room temperature conditions. The values used for numerical simulations, their applicability and the corresponding literature sources are given in detail in Table 1. As illustrated in Table 1, the analyzed glasses have radically different values of *α* and *dn*/*dT* at room temperature (293 K): for example, for fused silica (SiO_2_), *α* is an order of magnitude smaller than *dn*/*dT*, while for tellurite glass (TeO_2_–WO_3_–La_2_O_3_ or TWL), *dn*/*dT* is negative and its absolute value is comparable to that of SiO_2_. For chalcogenide glasses (As_2_S_3_ and As_2_Se_3_), *α* is approximately similar, being several times higher than for GeO_2_ and TWL, and almost 50 times higher than for regular SiO_2_; conversely, their *dn*/*dT* values differ drastically, as it is close to zero for As_2_S_3_ and for As_2_Se_3_ it is the highest among the considered materials. Consequently, studying WGM temperature sensors based on such a diverse selection of glasses is of certain interest.

**Figure 2:**
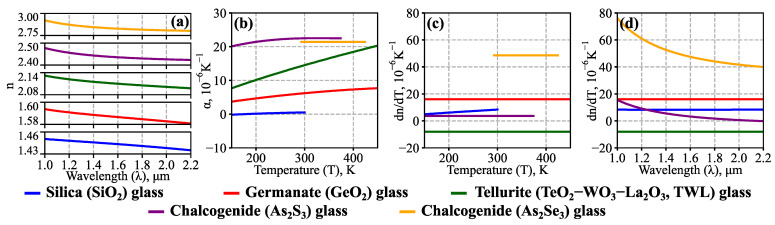
Optical and thermo-optical glass parameters used in numerical simulations (the corresponding literature sources are provided in Table 1). (**a**) Glass refractive index as a function of light wavelength *λ*. (**b**) Temperature dependence of thermal expansion coefficient *α*. (**c**,**d**) Thermo-optic coefficient *dn*/*dT* as a function of temperature *T* at *λ* = 1.55 μm (**c**) and as a function of *λ* at room temperature *T_0_* = 293 K (**d**).

We developed a numerical code for solving the characteristic Equation (1) taking into account glass dispersion (2) and thermal effects (4,5); high-order approximations were utilized iteratively to localize the roots [49]. The resonant wavelength shift Δ*λ* as a function of temperature change Δ*T* was calculated from the obtained data. Previously, we employed a similar approach for tellurite microspheres [36]; however, the present work accounts for glass parameter functions *α*(*T*) and *dn*(*T*,*λ*)/*dT*, which are plotted in Figure 2. The spatial distribution of Δ*T* was assumed to be homogeneous, as only stationary measurements of the ambient temperature were considered. The obtained values of Δ*λ* were used to calculate the sensitivity of the device *dλ*/*dT*.

## 3. Results

### 3.1. Temperature Sensitivity of Same-Sized Microspheres

**Figure 3:**
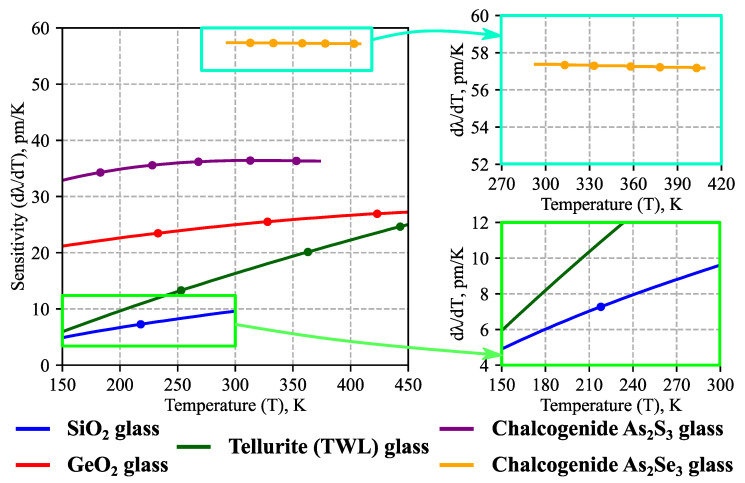
Calculated temperature sensitivities *dλ*/*dT* of spherical microresonators based on different glasses as a function of ambient temperature. The data are shown only for TE fundamental WGMs that are the closest to *λ* = 1.55 μm for the corresponding *T*; line segments between dots denote different operating WGMs. Room temperature (*T_0_* = 293 K) microsphere radius is *R_0_* = 100 μm.

We used the developed approach to study the temperature sensitivity *dλ*/*dT* of microresonators based on the considered glasses. The calculations were performed in a wide parameter range: 150 K ≤ *T* ≤ 450 K, 20 μm ≤ *R_0_* ≤ 200 μm (Section 3.2), 1.0 μm ≤ *λ* ≤ 2.2 μm (Section 3.3); the ambient temperature was additionally constrained by the availability of the specific glass data (see Table 1 and Figure 2b,c for clarification). The simulation results for same-sized (*R_0_* = 100 μm) microsensors made of different materials are shown in Figure 3; the data are plotted here only for the fundamental TE modes that are the closest to *λ* = 1.55 μm at the corresponding temperature.

As illustrated in Figure 3, the highest temperature sensitivity was achieved for the chalcogenide microresonators: 57 pm/K for As_2_Se_3_ and 37 pm/K for As_2_S_3_ at room temperature. This is significantly more than for the microspheres made of silica glass (9.4 pm/K) or of chalcogenide glass with different chemical composition (Ge_20_Ga_5_Sb_10_S_65_, 28 pm/K [37]). Moreover, the values of *dλ*/*dT* for As_2_S_3_ and As_2_Se_3_ are almost constant at high temperatures; this may be a convenient quality for microsensor calibration.

It is worth noting that the results for silica glass microspheres were in good agreement with experimental measurements [50], thus validating our theoretical model. In [50], the obtained sensitivity at cryogenic temperatures (120 K ≤ *T* ≤ 293 K) was 5.9 pm/K at 150 K, 7.8 pm/K at 200 K, and 10.7 pm/K at 293 K. The blue curve in Figure 3 illustrates the results of theoretical calculations for silica glass microresonators obtained in the present work: 4.9 pm/K at *T* = 150 K, 6.7 pm/K at *T* = 200 K, and 9.4 pm/K at room temperature.

The results shown in Figure 3 are only for fundamental TE modes of the same-sized, ideally spherical microresonators; in a real experiment, however, imperfections in microresonator dimensions are inevitable. Moreover, it is usually impossible to determine WGM polarization and structure from spectral data (an example of real-world Δ*λ* measurements can be found in [36]).

To account for these practically important factors, we calculated the temperature sensitivity *dλ*/*dT* for microsphere eigenmodes with different indices and polarizations. The WGMs with *q* ≥ 1 and *l* ≤ |*m*| were examined. In the considered setup, the highest excitation coefficient is achieved for “equatorially” localized WGMs, as its value is proportional to field overlap integral. Such modes have a relatively small number of radial and “meridional” variations (see Figure 1b–g), hence we limited our study to *q* ≤ 10, *l* − |*m*| ≤ 10. The numerical simulations showed that, for microspheres with *R_0_* = 100 μm, the relative variations in temperature sensitivity for WGMs with different structures and polarizations did not surpass 0.3% at room temperature. This quality is crucial for the development of microsensors, as it ensures the same *dλ*/*dT* for all WGMs near a specific wavelength.

**Figure 4:**
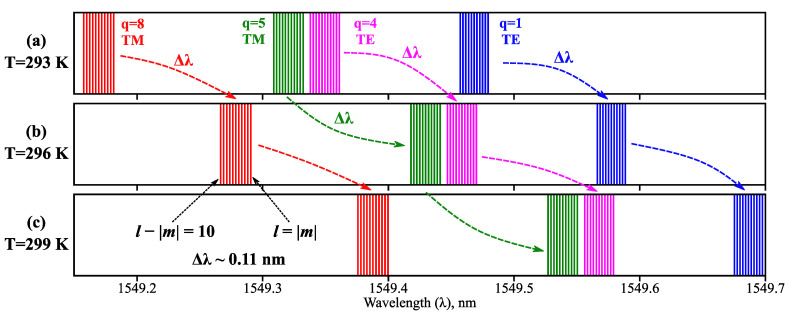
WGM resonant wavelengths (marked with vertical lines) calculated at different ambient temperatures: (**a**) *T* = 293 K, (**b**) *T* = 296 K, (**c**) *T* = 299 K. Modes with identical radial indices *q* and polarizations are denoted by lines of the same color; arrows indicate mode drift with changes in *T*. Data are shown for As_2_S_3_ microsphere with *R_0_* = 100 μm, 1 ≤ *q* ≤ 10, 0 ≤ *l* − |*m*| ≤ 10; shape deformation parameter *η* = 7 × 10^−4^. Total wavelength range is roughly a third of the fundamental WGM distance.

Additionally, we assessed possible sensitivity inconsistencies among different WGMs of a microsphere with minor shape imperfections. Figure 4 illustrates a few resonant frequencies of a slightly deformed microresonator at different temperatures; here the small shape deviation (*η* = 7 × 10^−4^) has broken the spherical symmetry of the system, thus lifting the mode degeneracy by *m* as shown in Equation (3). According to our calculations, to keep the relative variations in *dλ*/*dT* below 0.1% across WGMs with *l* − |*m*| ≤ 10 for a fixed deformed geometry (initial microsphere radius *R_0_* = 100 μm), the deformation parameter *η* must not exceed 0.03 for SiO_2_ glass and 0.06 for As_2_Se_3_ glass. The obtained results demonstrate high robustness of the proposed microsensor design to various manufacture imperfections, which is an important quality from a practical standpoint.

### 3.2. Temperature Sensitivity of Different-Sized Microspheres

**Figure 5:**
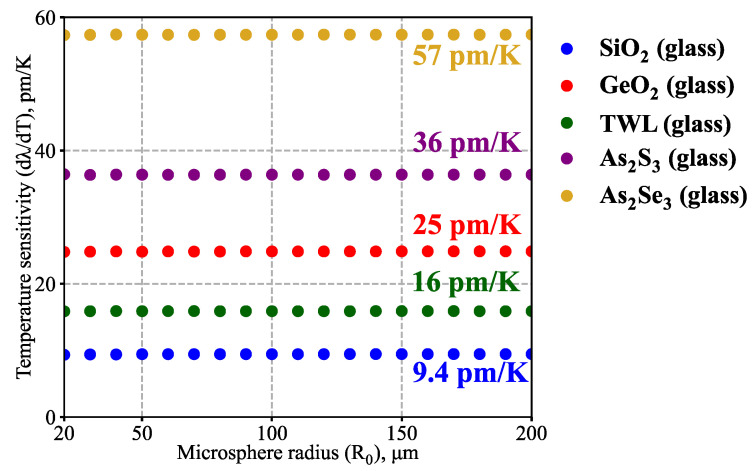
Numerically simulated temperature sensitivity *dλ*/*dT* at room temperature (*T_0_* = 293 K) as a function of microsphere radius *R_0_*. Results are shown for fundamental TE modes near *λ* = 1.55 μm.

Next, we analyzed the temperature sensitivity of microspheres with different *R_0_*: 20 μm ≤ *R_0_* ≤ 200 μm. The results are shown in Figure 5 at room temperature for microspheres made of the considered types of glasses (only fundamental TE modes are shown for the reasons mentioned above). Note that *dλ*/*dT* was almost independent of microsphere radius; according to our simulations, its relative variations did not exceed 1% in the examined parameter range.

**Figure 6:**
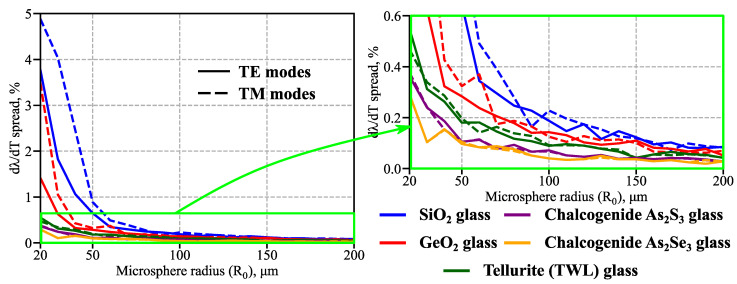
Calculated temperature sensitivity spread (maximum relative variation in *dλ/dT*) across WGMs with radial indices 1 ≤ *q* ≤ 10 as a function of microsphere radius *R_0_* for TE (solid lines) and TM (dashed lines) polarizations. Results are shown for *T* = *T_0_* = 293 K, *λ* = 1.55 μm.

Further, we thoroughly studied the temperature sensitivity of WGMs with various structures and polarizations for microspheres of different radii. We found *dλ*/*dT* to be almost independent of the system parameters: for 20 μm ≤ *R_0_* ≤ 200 μm, the largest spread of *dλ*/*dT* values across the modes with 1 ≤ *q* ≤ 10 was 5% (for SiO_2_ microsensors with *R_0_* = 20 μm). Microspheres with a larger *R_0_* exhibited significantly smaller temperature sensitivity variations: for *R_0_* ≥ 50 μm, the spread was less than 1% for silica microspheres and was less than 0.2% for tellurite and chalcogenide glass microspheres, as illustrated in detail in Figure 6.

As mentioned above, the weak sensitivity dependence on WGM structure is a crucial quality of microresonator-based temperature microsensors. Additionally, the results shown in Figure 5 and Figure 6 underline the insignificant role of the microsphere size, which was also experimentally verified for silica glass microcavities in [50]. Together with the weak constraints on spherical shape imperfections analyzed in Section 3.1, these qualities highlight the practical robustness of such WGM-based temperature microsensors.

The fact that temperature sensitivity is independent of microresonator shape and radius, as well as of mode structure and polarization, can be derived analytically from the approximate resonance condition:

(6) $$m\cdot \lambda =2\pi \cdot R\left(T\right)\cdot n_{eff}$$

where *n_eff_* is the effective refractive index of the mode, which is, strictly speaking, a function of mode structure and resonator configuration. A small temperature variation in *dT* will cause the corresponding resonant wavelength shift *dλ*. Since the same WGM is used for measurements, *m* = *const*. Therefore, Equations (4)–(6) can be rewritten as follows:

(7) $$d\lambda =\frac{2\pi }{m}\left(n_{eff}\cdot dR+R\left(T\right)\cdot dn\left(T\right)\;\right)$$

(8) $$d\lambda \approx \lambda \left(\alpha \left(T\right)+\frac{1}{n_{eff}}\cdot \frac{dn}{dT}\left(T,\;\lambda \right)\;\right)\cdot dT.$$

Since *n_eff_* rather weakly depends on microsphere radius, mode indices and light wavelength, Equation (8) directly shows that the sensitivity *dλ*/*dT* should be almost independent of these parameters. This feature is exceptionally convenient for the temperature microsensor design based on thermo-optical WGM shifts. However, as Equation (6) is written in the geometrical optics approximation, which assumes that the light propagates along the microsphere circumference, it becomes less accurate for smaller radii. For example, the WGM temperature sensitivity of bottle microresonators with diameters of 5–6 μm was found to change with the microcavity size in contrast to the results of Equation (8) [51]. The applicability of Equation (8) also explains the larger *dλ*/*dT* spread for lower *R_0_*.

### 3.3. Spectral Dependence of Temperature Sensitivity

**Figure 7:**
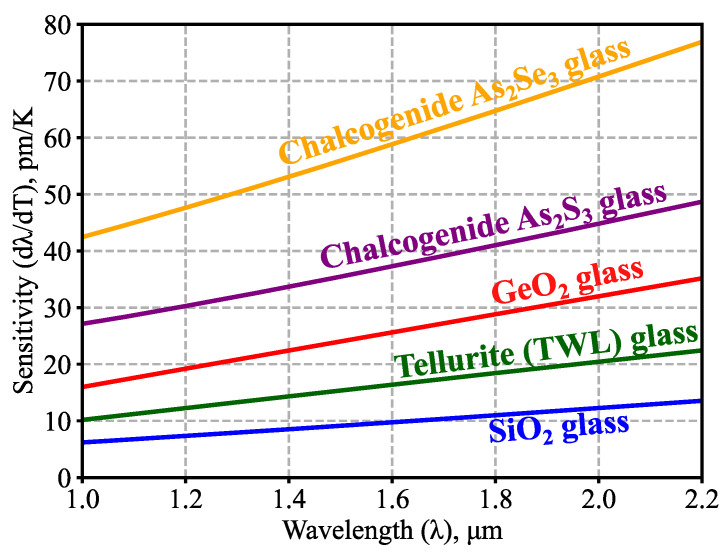
Calculated WGM temperature sensitivity as a function of the wavelength of light. The data are shown for room temperature conditions (*T_0_* = 293 K) for microspheres with *R_0_* = 100 μm.

Besides the telecommunication range considered above, measurements with other light wavelengths may also be of practical interest. For example, cheap broadband Yb and Tm fiber laser sources are available near 1 μm and 2 μm, respectively. Therefore, we also investigated the wavelength dependence of temperature sensitivity for 1.0 μm ≤ *λ* ≤ 2.2 μm. The results of the calculations are shown in Figure 7 at room temperature for glass microspheres with *R_0_* = 100 μm; it is clear that *dλ*/*dT* grows with an increase in *λ*; this tendency can be explained using the previously derived Equation (8), which shows that *dλ/dT* should be proportional to *λ* if we neglect the spectral dependence of the thermo-optic coefficient. As before, chalcogenide microsensors are of significant interest owing to their high sensitivity.

## 4. Discussion

We performed a systematic numerical analysis of highly sensitive spherical temperature microsensors with a realistic design based on both standard silica telecommunication glass fibers and special soft glass fibers (GeO_2_, tellurite, As_2_S_3_, and As_2_Se_3_), including commercially available ones. The operation of the considered thermometers relies on the effect of the thermo-optical whispering gallery mode frequency shift caused by changes in the ambient temperature. We calculated the sensitivity *dλ*/*dT* of the examined microsensors in a wide parameter range; the spectral and temperature dependence of the properties of glasses were taken into account. The highest *dλ*/*dT* was achieved for chalcogenide As_2_Se_3_ and As_2_S_3_ microspheres: 57 pm/K and 36 pm/K for measurements at room temperature near the light wavelength *λ* = 1.55 μm, respectively. These values are several times higher than for regular fused silica microspheres (9.4 pm/K) and higher than for a microsphere made of customized Ge_20_Ga_5_Sb_10_S_65_ chalcogenide glass (28 pm/K), which was examined in [37]. The results of the numerical simulations for SiO_2_ microspheres were in good agreement with the experimental data in a wide temperature range reported in [50].

Importantly, the sensitivity was almost independent of the microresonator size, WGM structure and polarization; this quality is of practical value as it shows the robustness of the considered sensor design. A simple analytical explanation of this fact was provided in terms of approximate resonance condition. We found that the variations in temperature sensitivity among different WGMs decreased for larger microsphere radii *R_0_*: *R_0_* ≥ 50 μm guaranteed less than 0.1% relative spread for chalcogenide microresonators. Additionally, we assessed possible *dλ*/*dT* inconsistencies across WGMs of different structures: the effect was found to be negligible. As the considered chalcogenide fibers are commercially available, glass microsphere fabrication methods are well known and fairly simple, and the size and shape requirements are minimal, such microsensors can become relatively cheap, robust devices for high-accuracy, on-site temperature measurements. Notably, *dλ/dT* is also independent of the WGM Q-factor, which defines only the width of the resonance rather than the position itself. In the considered measurement scheme, the minimal temperature resolution is determined by the wavelength resolution of the used optical spectrum analyzer, which can be of the order of 0.01 nm. Alternatively, a tunable, narrow-band, continuous-wave (CW) laser operating in the sweeping mode may be utilized as a light source to increase the accuracy. In this case, a photodetector and an oscilloscope are needed to diagnose the output radiation. This CW-based scheme can improve the temperature resolution by several orders of magnitude.

The present work may serve as an effective guide for the development of highly accurate robust thermo-optical sensors that are made of commercially available glass fibers and, simultaneously, take advantage of the well-developed telecommunication base.

## Data Availability

Data underlying the results presented in this article may be obtained from the authors upon reasonable request.
